# Supplementary material for: The genome sequence of the muscid fly, Hydrotaea similis Meade, 1887 (Diptera: Muscidae)
Source: Wellcome Open Res. 2026 Aug 8;11:399. Originally published 2026 Jun 27. [Version 2] doi: 10.12688/wellcomeopenres.26885.2 (PMC13560591; doi:10.12688/wellcomeopenres.26885.2)
Supplement: Peer review history

# Reviewer report: Reviewer response for version 2

**DOI:** 10.21956/wellcomeopenres.30241.r163713

**Published:** 2026-08-10

**Authors:** Shilpa Kapoor

**Affiliations:** 1 The University of Melbourne, Parkville, Australia

Thank you to the authors for revising the article and addressing the comments. I am satisfied with the revisions and have no further comments.

Are sufficient details of methods and materials provided to allow replication by others?

Partly

Is the rationale for creating the dataset(s) clearly described?

Yes

Are the datasets clearly presented in a useable and accessible format?

Yes

Are the protocols appropriate and is the work technically sound?

Yes

Reviewer Expertise:

Genomics, Bioinformatics

I confirm that I have read this submission and believe that I have an appropriate level of expertise to confirm that it is of an acceptable scientific standard.

# Reviewer report: Reviewer response for version 1

**DOI:** 10.21956/wellcomeopenres.29604.r161495

**Published:** 2026-08-04

**Authors:** Shilpa Kapoor

**Affiliations:** 1 The University of Melbourne, Parkville, Australia

This Data Note presents a high-quality genome assembly for the muscid fly *Hydrotaea similis*, generated as part of the Darwin Tree of Life initiative. The authors provide a chromosomal-level assembly from a single specimen collected in Wytham Woods, UK. The study combines PacBio HiFi sequencing, Hi-C scaffolding and modern quality assessment approaches. The final primary assembly spans 884.66 Mb, with 90.83% of the sequence assigned to five chromosomal pseudomolecules and excellent quality metrics including BUSCO completeness of 99.4% and Merqury QV of 57.5. The methods are clearly described, the assembly statistics are transparently reported, and the resulting dataset will be valuable for comparative genomics, Muscidae phylogeny, and studies of forensic entomology. Overall, this is a technically sound and useful genomic resource that is suitable for publication after the following revisions.

 Minor comments:

 1) The most important issue identified is an inconsistency concerning the sex of the sequenced specimen.In the Abstract and Methods sections, the specimen is described as a female. However, the Background section states: “We present a chromosomally complete genome sequence for *Hydrotaea similis*, based on a single male specimen from Wytham Woods…” Later, the Methods clearly describe the specimen as an adult female. This discrepancy should be corrected throughout the manuscript. 

 2) The authors state that sex chromosomes could not be identified because data from the heterogametic sex were unavailable. Assuming the specimen is indeed female, this explanation is reasonable and consistent with the frequent sex chromosome transitions reported in Diptera. Once the sex discrepancy noted above is corrected, this discussion should be reviewed to ensure the rationale remains internally consistent.

 3) GenomeScope estimated a haploid genome size of approximately 655 Mb, whereas the primary assembly spans 884.66 Mb and the alternate haplotype spans 852.78 Mb. The manuscript reports these values correctly, but a brief explanation for the difference would improve clarity. The relatively high heterozygosity estimate of 2.09% may contribute to assembly inflation and haplotype separation. A short explanatory sentence would help readers unfamiliar with phased or diploid-aware assemblies.

 4) On page 3, “In Britain, is considered a frequent species…” should be replaced with “In Britain, it is considered a frequent species…”

 5) Page 2, Grant information - Change “Andrzej Grzywacz received supported from” to “Andrzej Grzywacz received support from” or “was supported by”.

 6) Page 4, Use consistent manufacturer styling: “NanoDrop” rather than “Nanodrop”; and “Thermo Fisher Scientific” rather than “ThermoFisher Scientific”.

 7) Pages 3-4, Replace “see the protocol” with a direct, formal citation to the exact protocol.

 8) Page 10, Data availability - Use “RNA-seq” consistently rather than “RNA-Seq”.

 9) Italicise all genus and species names consistently in keywords and data availability statement.

 10) Use “chromosome-level” or justify “chromosomally complete”.

Are sufficient details of methods and materials provided to allow replication by others?

Partly

Is the rationale for creating the dataset(s) clearly described?

Yes

Are the datasets clearly presented in a useable and accessible format?

Yes

Are the protocols appropriate and is the work technically sound?

Yes

Reviewer Expertise:

Genomics, Bioinformatics

I confirm that I have read this submission and believe that I have an appropriate level of expertise to confirm that it is of an acceptable scientific standard, however I have significant reservations, as outlined above.

# Response

**Published:** 2026-08-04

**Authors:** Tree of Life Team Sanger

**Affiliations:** Wellcome Trust Sanger Institute, UK

Thank you for reviewing this article.   This is a female fly, and we have corrected the error in the Background section. We have also made other corrections that you requested.   Please note that the keywords cannot be italicised.   The individual protocols are linked directly in the Methods section. We have not included them in the reference list because the Wellcome Open Research guidelines state that “Web links, URLs, and links to the authors’ own websites should be included as hyperlinks within the main body of the article, and not as references.” We have therefore retained direct hyperlinks to the relevant protocols rather than adding separate reference-list entries. https://wellcomeopenresearch.org/for-authors/article-guidelines/preparing-a-data-note/#ref   The Tree of Life team

# Reviewer report: Reviewer response for version 1

**DOI:** 10.21956/wellcomeopenres.29604.r161492

**Published:** 2026-08-03

**Authors:** Ezra M. Bailey

**Affiliations:** 1 Entomology and Plant Pathology, North Carolina State University, Raleigh, North Carolina, USA

Here, the authors present a high quality assembly of an abundant and widely distributed insect which is part of a phylogenetically contentious grade. The authors have written a clear, concise report with valuable natural history information. Methods are robust, resulting in an excellent resource for phylogenomic and comparative genomic study. Perhaps the variation of chromosome count in Muscidae can be added to the background to further emphasize the utility of this chromosome-scale assembly for comparative analyses; though the limitation of sex chromosome identification and reference to Vicoso and Bachtrog in the report make sense. The article is scientifically sound; the thoughtfully prepared resource will be very beneficial to those studying the muscoid grade and other calyptrates.

Are sufficient details of methods and materials provided to allow replication by others?

Yes

Is the rationale for creating the dataset(s) clearly described?

Yes

Are the datasets clearly presented in a useable and accessible format?

Yes

Are the protocols appropriate and is the work technically sound?

Yes

Reviewer Expertise:

genomics, phylogenomics, Calyptratae

I confirm that I have read this submission and believe that I have an appropriate level of expertise to confirm that it is of an acceptable scientific standard.
